# In this month’s Bulletin

**DOI:** 10.2471/BLT.21.000921

**Published:** 2021-09-01

In the editorial section, Delia Enria et al. (610) call for research into the effects of each public health and social measure used by governments in the COVID-19 response. Saverio Bellizzi et al. (611) describe a national SARS-CoV-2 vaccination programme that includes migrants and refugees.

In the news section, Tatum Anderson (614–615) reports on the need for investment in mental health services in the context of the COVID-19 pandemic. Kazuto Kato talks to Gary Humphreys (616–617) about the ethical and societal challenges posed by editing of the human genome. 

## African Region 

### Inequalities in immunization coverage 

Katherine Kirkby et al. (627–639) analyse subnational completion of diptheria-tetanus-pertussis courses.

## Malawi 

### Healthcare for men with HIV 

Kathryn Dovel et al. (618–626) count visits and evaluate services.

## Thailand

### Preventing antimicrobial resistance 

Nithima Sumpradit et al. (661–673) describe implementation of the national strategic plan.

## United Kingdom of Great Britain and Northern Ireland 

### Accessibility and efficiency of mental health services 

Shanquan Chen & Rudolf N Cardinal (674–679) describe the investments needed.

## United States of America

### Racial differences in pandemic outcomes 

Don E Willis & Pearl A McElfish (680–681) note disproportionate morbidity among the Marshall Islands diaspora.

## Global

### Treating stroke in low- and middle-income countries 

Joseph Yaria et al. (640–652) review the evidence for effectiveness of guidelines.

### Treating stroke in all settings

Ignacio Neumann et al. (653–660) argue for global access to direct oral anticoagulants.

### The intersection of gender and emerging infectious diseases

Lynn Lieberman Lawry et al. (682–684) propose a framework for programme design.

